# Light Spectrum, Intensity, and Photoperiod Are Key for Production as Well as Speed Breeding of Spring Wheat in Indoor Farming

**DOI:** 10.1002/pei3.70085

**Published:** 2025-09-10

**Authors:** Jinglai Li, Yuqi Zhang, Ruifeng Cheng, Tao Li

**Affiliations:** ^1^ Institute of Environment and Sustainable Development in Agriculture Chinese Academy of Agricultural Sciences Beijing China

**Keywords:** indoor farming, light intensity, light spectrum, photoperiod, speed breeding, wheat

## Abstract

Wheat ( *Triticum aestivum* L.) is the 3rd largest food crop worldwide. Growing wheat in indoor farming is a possible option for increasing future production and to facilitate speed breeding. Light is one of the most important environmental factors, but the light recipe for growing wheat in indoor farming is not well researched. We aimed to investigate the effects of light spectrum (including color temperature), intensity, photoperiod, and pattern on spring wheat growth, flowering time, and yield. Under full‐spectrum white LEDs, a color temperature of 3500 K caused anthesis to be 4 days earlier and increased yield by 13% compared with 4500 K at a light intensity of 500 μmol m^−2^ s^−1^. At a light intensity of 700 μmol m^−2^ s^−1^, plants entered anthesis 3–10 days earlier compared to those under 300–500 μmol m^−2^ s^−1^, and achieved the highest yield. Continuous light caused 3–4 days earlier anthesis but caused a 13% yield reduction compared to a photoperiod of 22 h at the same daily light integral (~40 mol m^−2^ day^−1^). Under a dynamic light intensity pattern (~300 μmol m^−2^ s^−1^ from emerging to tillering; ~700 μmol m^−2^ s^−1^ from elongation to heading; ~400 μmol m^−2^ s^−1^ after flowering), flowering was triggered as early as under constant 700 μmol m^−2^ s^−1^ while saving ~30% of light input. We suggest that both a constant and a dynamic lighting recipe can be used to grow spring wheat indoors, potentially leading to more than seven generations per year.

## 1 Introduction

With increasing concerns about the need for high crop productivity at times of climate change, indoor farming (also known as vertical farm system or plant factory) offers a new strategy to address agricultural challenges. Indoor farming enables year‐round efficient high quality crop production by optimizing environmental control (Yano et al. 2023). To date, indoor farming is mostly used for commercial production of leafy vegetables (Wong et al. 2020) and dwarf plants (Zhang et al. 2022). However, it also has the potential for cereal production, including rice (Ren et al. 2023), wheat (Guo et al. 2023), and soybean (Righini et al. 2024). Wheat ( *Triticum aestivum* L.) is the 3rd largest food crop worldwide, accounting for 20% of calories in the human diet (Safdar et al. 2023). According to Asseng et al. (2020), wheat cultivated in ten‐layer racks under fully controlled environment can produce 220–600× more yield than the current world average annual wheat yield in open field production. Besides, indoor farming can facilitate crop breeding, as—unlike open fields—it allows for multiple generations per year (Kabade et al. 2024; Xu et al. 2022). In indoor farming, environmental control is vital for optimal plant growth and production, and the light environment is among the most important environmental factors. However, the strategy of controlling the light environment for fast breeding and production of wheat is still underdeveloped.

The light environment includes light intensity, photoperiod, daily light integral (DLI), light quality (i.e., spectral distribution), as well as light intensity pattern. As for light quality, different combinations of red, blue, and white LEDs have mostly been used to grow wheat indoors (Guo et al. 2023; Jia et al. 2021; Liu et al. 2023). A previous study showed that a higher red light fraction (~55%) promoted biomass accumulation in winter wheat at the seedling stage compared with a low red light fraction (20%) (Li et al. 2022). In indoor lighting, color temperature is used to describe the “warmness” of light; for this, the temperature (in Kelvin, K) at which an ideal black body would emit radiation of the same color as an LED is used. For example, a 2700 K light source is enriched in red light, while a 6500 K source is enriched in blue. Compared with cool‐white LEDs (> 4500 K), warm‐white LEDs (2500–3500 K) increased the biomass in spinach (Burattini et al. 2017) and lettuce (Vereshchagin et al. 2024), but it is unclear how color temperature affects wheat. As for light intensity, increasing light intensity (from 100 to 380 μmol m^−2^ s^−1^ in spring wheat and from 250 to 500 μmol m^−2^ s^−1^ in winter wheat) increased the photosynthesis rate, number of tillers, biomass, and yield (Page and Feller 2016). Especially in spring wheat, the increase in growth light intensity induced early flowering (Page and Feller 2016). Previously, a photosynthetic photon flux density (PPFD) of 450–500 μmol m^−2^ s^−1^ was recommended for speed breeding in wheat (Ghosh et al. 2018). However, with the advent of high‐power LEDs, it is worthwhile investigating the effects of higher intensities (e.g., 600–900 μmol m^−2^ s^−1^). Besides, as crops have different demands for light sum at different growth stages, tailoring the light recipe to different developmental stages may further optimize light use efficiency. Cha et al. (2023), Chhabra et al. (2024) and Watson et al. (2018) established speed breeding systems for wheat at photoperiods of 22 h, leading to 5–6 generations per year. However, it remains to be investigated whether a further extension of the photoperiod (> 22 h) can further shorten the generational cycle of wheat.

This study aimed at developing a lighting strategy that can facilitate speed breeding as well as promote the production of spring wheat in indoor farming. We explored the effects of light spectrum, light color temperature, light intensity, photoperiod as well as light pattern on wheat growth, development, leaf photosynthesis, yield, and seed quality. Concluding, we suggest two lighting recipes that can achieve more than seven generations per year for spring wheat cultivation in indoor farming.

## 2 Materials and Methods

### 2.1 Plant Material and Growth Conditions

Spring wheat ( *Triticum aestivum* L., cv. “Jinqiang 12”) seeds were washed in tap water for 5 min to remove dust and impurities and then immersed in 5% hydrogen peroxide for 30 min. After thoroughly rinsing them in distilled water, the seeds were pre‐germinated on moist germination paper at 25°C for 24 h in darkness until radicles were > 1 cm (Guo et al. 2023).

In the climate room, there were eight compartments of 1.2 m (length) × 1.2 m (width) × 1.2 m (height) each. LED lamps in each compartment were adjusted regularly such that the distance between lamps and the top of plants was 30 cm. PPFD and light spectrum were monitored with a spectroradiometer (Avaspec‐2048CL, Avates, Apeldoorn, Netherlands). An opaque white plastic reflective film was placed in front of each compartment to prevent light pollution from adjacent treatments. Unless stated, wheat grew at a temperature of 24/18°C (day/night), relative humidity of 65% ± 5%, and CO_2_ partial pressure of 700 ± 50 μbar.

In the light quality experiment, pre‐germinated seeds were sown into sponge cubes (2 × 2 × 2 cm). After 3rd leaves appeared (10 days after sowing), plants were transplanted into hydroponic tanks, with 300 plants in each tank (L120 × W90 × H10 cm), at a density of 225 plants m^−2^. The nutrient solution was completely refreshed once a week, and the hydroponic circulation system ran for 2 h per day. As we observed a relatively slow growth rate under hydroponic cultivation, we used substrate cultivation instead in the following experiments.

In the light intensity, color temperature, and light regime experiments, pre‐germinated seeds were sown in nursery plates containing peat soil (2 × 2 × 2 cm) and transferred to the same growth condition as hydroponic cultivation. After 3rd leaves appeared (10 days after sowing), they were transplanted into pots (9 plants per pot, 13 × 13 × 10 cm, 1.3 L) containing peat moss, perlite, and vermiculite (3:1:1), with a density of 225 plants m^−2^. Plants were irrigated via a drip irrigation system with a nutrient solution once per day.

The modified Hoagland nutrient solution was used in both the hydroponic and substrate systems. The recipe was as follows: Ca(NO_3_)_2_·4H_2_O, 1417 mg L^−1^; KNO_3_, 910 mg L^−1^; NH_4_H_2_PO_4_, 172 mg L^−1^; MgSO_4_·7H_2_O, 739 mg L^−1^; Fe EDTA, 45 mg L^−1^; MnSO_4_·H_2_O, 2.4 mg L^−1^; CuSO_4_·5H2O, 0.12 mg L^−1^; ZnSO_4_·7H_2_O, 0.33 mg L^−1^; (NH_4_)_6_Mo_7_O_24_·4H_2_O, 0.03 mg L^−1^; H_2_BO_3_, 4.29 mg L^−1^ (EC = 1.5–1.8 dS m^−1^; pH = 6.3–6.5).

### 2.2 Experimental Set Up

#### 2.2.1 Light Quality Experiment

**FIGURE 1:**
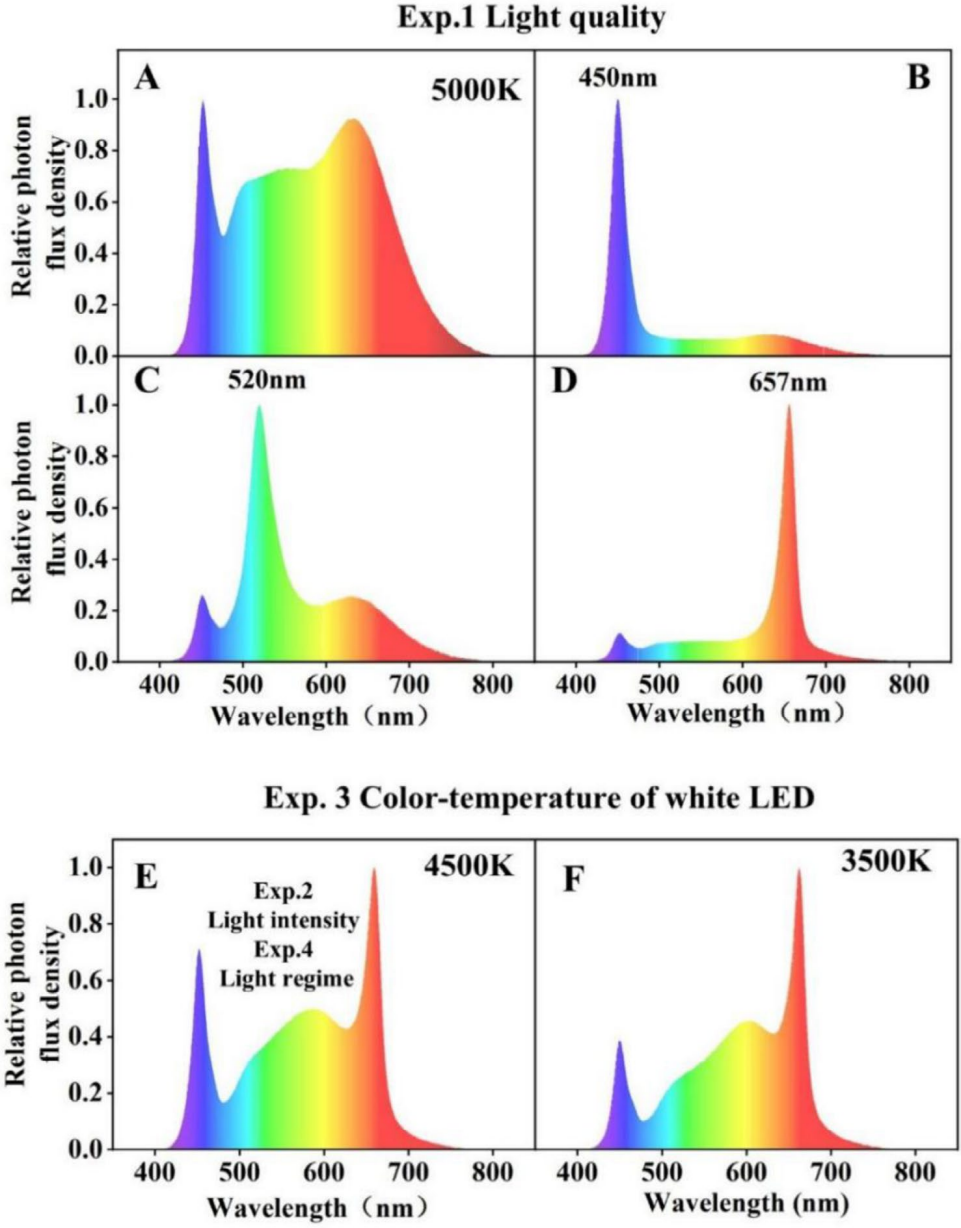
Relative photon flux density of growth light spectrum in different experiments. (A) spectrum of white LED panel with color temperature of 5000 K; (B–D) relative photon flux density of white LED plus blue LED (W + B), white LED plus green LED (W + G) and white LED plus red LED (W + R) panels. (E and F) spectrum of white LED panels with color temperatures of 4500 and 3500 K, respectively.

Wheat was grown under four different light quality treatments: white (W), white + green (W + G), white + blue (W + B), white + red (W + R) (Figure 1A–D; Table 1). PPFD above the plant canopy was kept at ~300 μmol m^−2^ s^−1^, by shifting LED panels upwards at regular intervals. Plants were grown in a hydroponic system at a photoperiod (day/night) of 20/4 h.

**TABLE 1:** Lighting setup in the light quality experiment.

Treatment	PPFD (μmol m^−2^ s^−1^)	Red (%)	Green (%)	Blue (%)
W	300	37	36	21
W + R	300	68	18	10
W + G	300	24	57	15
W + B	300	16	16	65

*Note:* Wheat was grown under four different light quality treatments: white (W), white + green (W + G), white + blue (W + B), white + red (W + R). Red light is defined as 600–700 nm, green light is defined as 500–600 nm, and blue light is defined as 400–500 nm.

#### 2.2.2 Light Intensity Experiment

Wheat was grown under four light intensities, namely 300, 500, 700, and 900 μmol m^−2^ s^−1^, with 4500 K full‐spectrum white LEDs (Figure 1E). Plants were grown in substrate cultivation at a photoperiod (day/night) of 22/2 h.

#### 2.2.3 Color Temperature Experiment

Plants were grown in substrate cultivation under white LEDs with two different color temperatures (4500 and 3500 K; Figure 1E,F; Table 2) at a photoperiod (day/night) of 22/2 h and PPFD of 500 μmol m^−2^ s^−1^.

**TABLE 2:** Lighting setup in color temperature experiment.

Treatment	PPFD (μmol m^−2^ s^−1^)	Red (%)	Green (%)	Blue (%)
3500 K	500	50	36	14
4500 K	500	41	38	21

*Note:* Red light wavelength was defined as 600–700 nm, green light wavelength was defined as 500–600 nm, and blue light wavelength was defined as 400–500 nm.

#### 2.2.4 Light Regime Experiment

Plants were grown in substrate cultivation under three different light regimes, with 4500 K full‐spectrum white LEDs (Figure 1E) and a similar daily light integral: (1) Control: 500 μmol m^−2^ s^−1^ PPFD at a photoperiod of 22 h; (2) Continuous: 458 μmol m^−2^ s^−1^ PPFD at a photoperiod of 24 h (continuous light); (3) Dynamic: different light intensities used during different developmental stages, at a photoperiod of 22 h (Table 3).

**TABLE 3:** Lighting setup in light regime experiment.

Treatment	Photo‐period (h)	PPFD (μmol m^−2^ s^−1^)			Planned average DLI (mol m^−2^)	Actual average DLI (mol m^−2^)
		Seedling emergence‐tillering	Elongation‐heading	Anthesis‐grain filling		
Control	22	500	500	500	39.6	39.6
Dynamic	22	300	720	400	39.6	38.7
Continuous	24	458	458	458	39.6	39.6

*Note:* Light was provided by full‐spectrum white LEDs with a color temperature of 4500 K. DLI, daily light integral. Planned average DLI was calculated based on the number of days that plants may need in different growing stages according to previous experiments, assuming no difference among treatments. Actual average DLI was recalculated based on the accurate dates needed in different growing stages.

#### 2.2.5 Early‐Harvesting Experiment

Wheat seeds can be categorized depending on their maturity: watery ripe, milky stage, soft dough, hard dough, and harvest ripe (Del Ponte et al. 2007). Plants were grown in substrate cultivation under white LEDs (Figure 1E) at a photoperiod (day/night) of 22/2 h and PPFD of 500 μmol m^−2^ s^−1^. Seeds from five different growth stages were randomly selected and dried at 38°C for 2 days until the moisture content was reduced to ~15%. The germination test was conducted three times with 50 seeds per growth stage. Seeds were first immersed in 5% hydrogen peroxide for 30 min and then rinsed fully with distilled water. Afterwards, seeds were germinated for 48 h on a wet germination paper at 25°C in darkness, and the germination rate was recorded.

### 2.3 Recording of Developmental Stage

The number of days between sowing and heading, flowering and harvest ripeness was recorded. When > 50% of plants in one pot entered a specific stage, it was recorded that all plants in this pot entered this stage.

### 2.4 Photosynthetic Gas Exchange Rate

At heading stage, leaf photosynthetic rate and stomatal conductance were measured using the LI‐6800 photosynthesis system (LI‐COR Biosciences, Lincoln, NE, USA), equipped with a leaf chamber fluorometer (LI‐COR, 6800‐01A, enclosed leaf area: 2 cm^2^). All measurements were performed at a leaf temperature of 25°C, leaf‐to‐air vapor pressure deficit of 0.7–1.0 kPa, flow rate of 500 μmol s^−1^ and CO_2_ partial pressure of 700 μbar. Irradiance was provided by a mixture of red (60%) and blue (40%) LEDs in the fluorometer. Peak intensities of red and blue LEDs were at wavelengths of 625 and 475 nm, respectively. After leaves were adapted in the leaf chamber for 15–20 min under the set light intensity (growth light intensity) until net photosynthesis rate reached steady‐state, gas exchange rate was recorded. Six plants per treatment were measured.

### 2.5 Chlorophyll Fluorescence

After leaves were first dark adapted (by enclosing them with aluminum foil) for 30 min, the minimum (*F*_o_) and maximum (*F*_m_) chlorophyll fluorescence were measured by a portable fluorescence spectrometer (Fluorpen FP110, MP100, Canada) to determine the maximum quantum efficiency (*F*_v_/*F*_m_) of photosystem II photochemistry.

### 2.6 Chlorophyll Index

Chlorophyll index was measured using an in situ plant multi‐pigment measuring instrument (MPM100, OPTI, America).

### 2.7 Plant Growth

At the mature stage, plants were randomly selected for growth analysis, including culm height, tiller number, and shoot dry weight. Leaves and internodes were counted from the bottom up. Internode 1 was the oldest internode, and the same numbering was used for leaves. Dry weight was recorded after plant material was oven‐dried at 70°C for 3 days. At the heading stage, flag leaf area (top leaf) was measured with a leaf area meter (Hai Zhuo, YMJ‐B, China). Six pots per treatment were randomly selected.

### 2.8 Yield Analysis

When wheat plants entered the harvest ripe stage, wheat ear number, grain number per ear, and thousand grain weight were measured. Yield was calculated as:

(1) $$Y=\frac{E*G*T*\mu }{1000}$$

where *Y* is yield (g m^−2^), *E* is spike number per plant (spike plant^−1^), *G* is grain number per spike (grain spike^−1^), *T* is thousand grain weight (g), and *μ* is planting density (225 plants m^−2^).

### 2.9 Seed Protein and Starch Content

After seeds were dried to a water content of ~15%, protein and starch contents were measured with a DA7200 multifunctional near infrared analyzer (Perten, IM‐9520, Sweden).

### 2.10 Statistical Analysis

Each experiment (light treatment) was conducted twice (resulting in two blocks), with individual plants treated as biological replicates. The number of replicates per measurement is specified in the figure captions. One‐way ANOVA in randomized blocks was performed followed by Duncan's test at the 95% confidence level to test for differences between treatments using IBM SPSS 23 (IBM Corp., Armonk, NY, United States). Figures were drawn using Origin 2018 (Origin, Origin Lab, Northampton, MA, USA) and R studio (Rstudio2022, Murray Hill, NJ, USA).

## 3 Results

### 3.1 Effects of Light Quality on Spring Wheat Growth and Development

**FIGURE 2:**
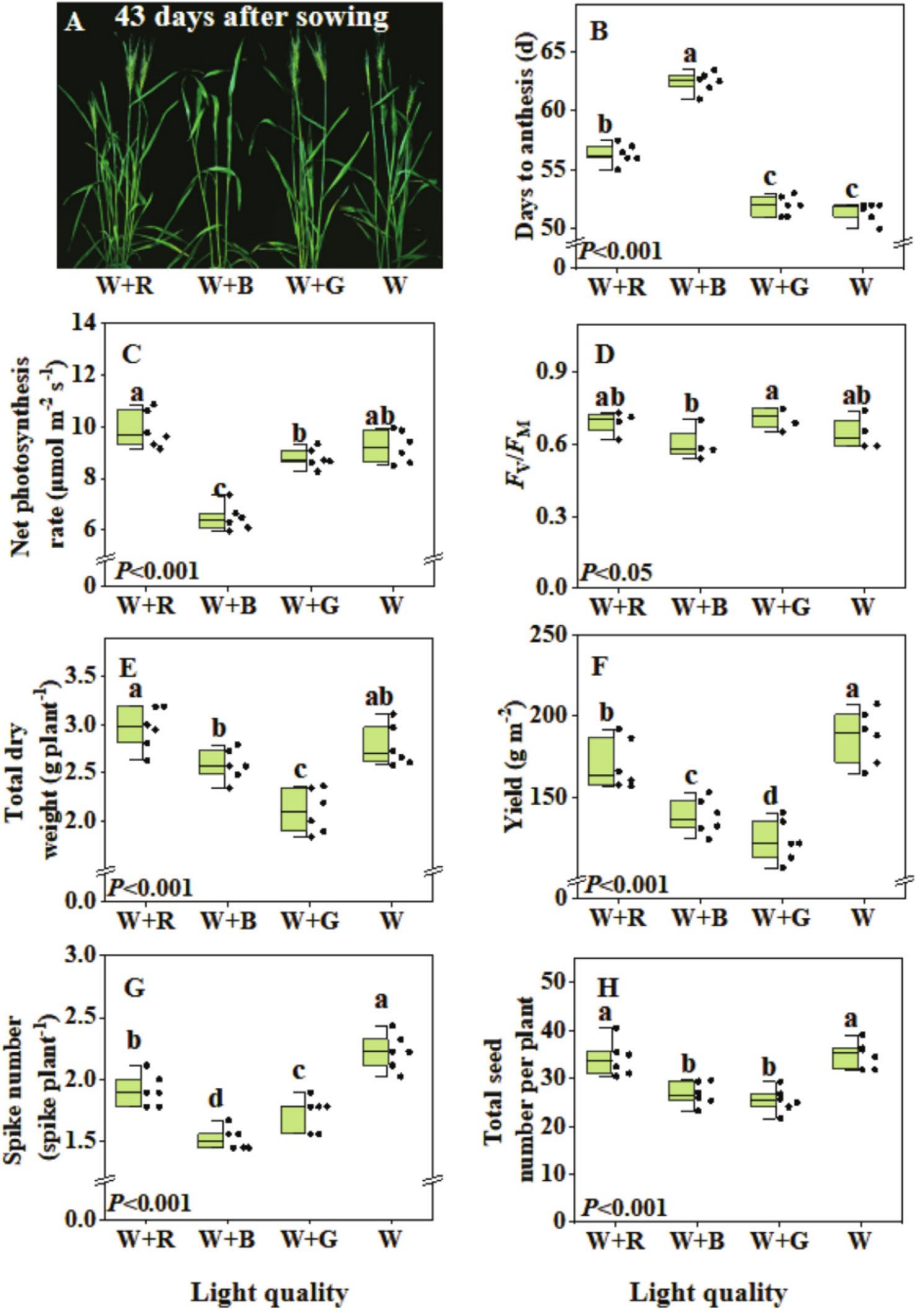
Effects of light quality on spring wheat growth and production. (A) Image of plants at 43 days after sowing; (B) days from sowing to anthesis (*n* = 6); (C) net photosynthesis rate at 300 μmol m^−2^ s^−1^ (*n* = 6); (D) maximum photochemical efficiency of photosystem II (*F*_v_/*F*_m_) (*n* = 6); (E) total dry weight (including stem and spike) (*n* = 6); (F) yield (*n* = 6); (G) spike number (*n* = 6); (H) total seed number per plant (*n* = 6). Wheat was grown under four different light quality treatments: White (W), white + green (W + G), white + blue (W + B), white + red (W + R) at PPFD of ~300 μmol m^−2^ s^−1^. Box plots indicate 0% (maximum), 25%, 50% (median), 75%, and 100% (minimum) and points show single data. Different letters represent significant differences between treatments according to Duncan's analysis at *p* = 0.05.

Light quality affected the growth and the flowering time of wheat (Figure 2A,B). W + B and W + R delayed flowering compared to the W + G and W treatments (Figure 2B). The net photosynthesis rate of W + R was 13% and 57% higher than that of W + G and W + B, respectively, but was not different from W (Figure 2C). W + B decreased *F*_v_/*F*_m_ compared to other treatments (Figure 2D). The W + B treatment increased flag leaf area and culm height compared to other treatments (Table S1). W and W + R increased total dry weight compared to other treatments (Figure 2E). The yield of W was 11%, 37%, and 53% higher than that of W + R, W + B, and W + G, respectively (Figure 2F). W increased spike number compared to other treatments (Figure 2G). Although under W and W + G, the number of grains per spike decreased compared to the other two treatments (Table S1), under W and W + R, the total seed number per plant was increased (Figure 2H). W + R and W + G decreased the thousand‐grain weight compared to the other two treatments (Table S1). The W + G treatment increased seed protein and decreased starch content compared to other treatments (Table S1).

### 3.2 Effects of Light Intensity on Spring Wheat Growth and Development

**FIGURE 3:**
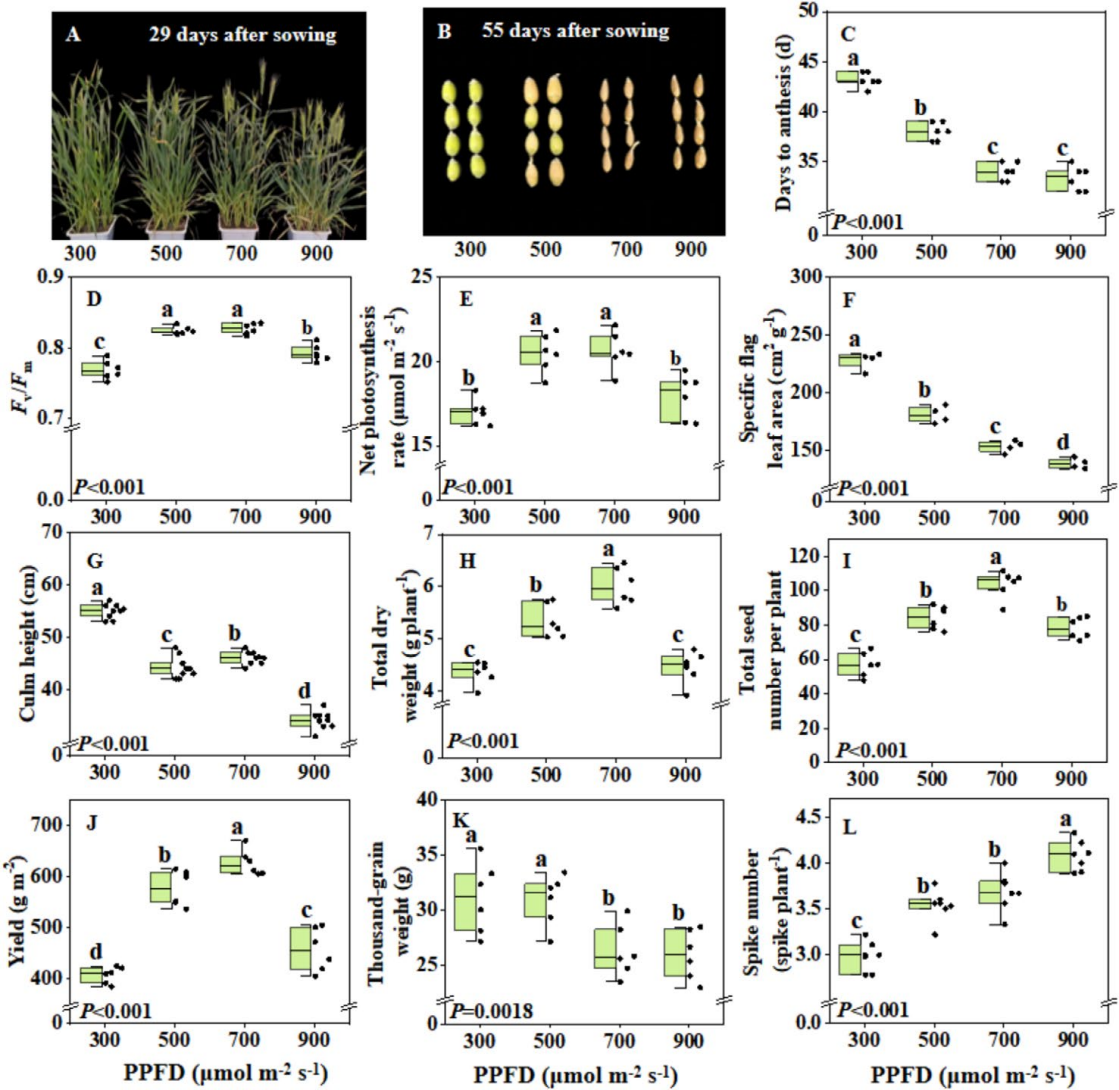
Effects of light intensity on spring wheat growth and production. (A) image of plants at 29 days after sowing; (B) image of seeds at 55 days after sowing; (C) days from sowing to anthesis (*n* = 6); (D) maximum photochemical efficiency of photosystem II (*F*_v_/*F*_m_) (*n* = 6); (E) net photosynthesis rate (at corresponding growth light intensity) (*n* = 6); (F) specific flag leaf area (*n* = 4); (G) culm height (*n* = 9); (H) total dry weight (including stem and spike) (*n* = 6); (I) total seed number per plant (*n* = 6); (J) wheat yield (*n* = 6); (K) thousand‐grain weight (*n* = 6); (L) spike number per plant (*n* = 6). Box plots indicate 0% (maximum), 25%, 50% (median), 75%, and 100% (minimum), and points show single data. Different letters represent significant differences between treatments according to Duncan's analysis at *p* = 0.05.

Images of plant growth and seed maturity at different growth stages were shown in Figure 3A,B. Wheat plants grown under 700 and 900 μmol m^−2^ s^−1^ flowered 5–10 days earlier than those under lower PPFD (Figure 3C). Net leaf photosynthesis rate and *F*_v_/*F*_m_ were highest under 500 and 700 μmol m^−2^ s^−1^, compared to the two other treatments (Figure 3D,E). Treatment with 300 μmol m^−2^ s^−1^ reduced dry matter partitioning to spikes, compared to higher PPFDs (Table S2). The increase in light intensity from 300 to 900 μmol m^−2^ s^−1^ continuously reduced the specific flag leaf area and culm height while increasing the spike number (Figure 3F,G,L). The increase in PPFD from 300 to 700 μmol m^−2^ s^−1^ led to a continued increase in total dry weight, total seed number, wheat yield and grain number per spike, but those were reduced under 900 μmol m^−2^ s^−1^ (Figure 3H–J; Table S2), suggesting an optimum at 700 μmol m^−2^ s^−1^ for these traits. When light intensity increased to 700 μmol m^−2^ s^−1^, thousand‐grain weight decreased significantly (Figure 3K). Besides, seed protein and starch content were only slightly affected by light intensity treatments (Table S2).

### 3.3 Effects of Light Regimes on Spring Wheat Growth and Development

**FIGURE 4:**
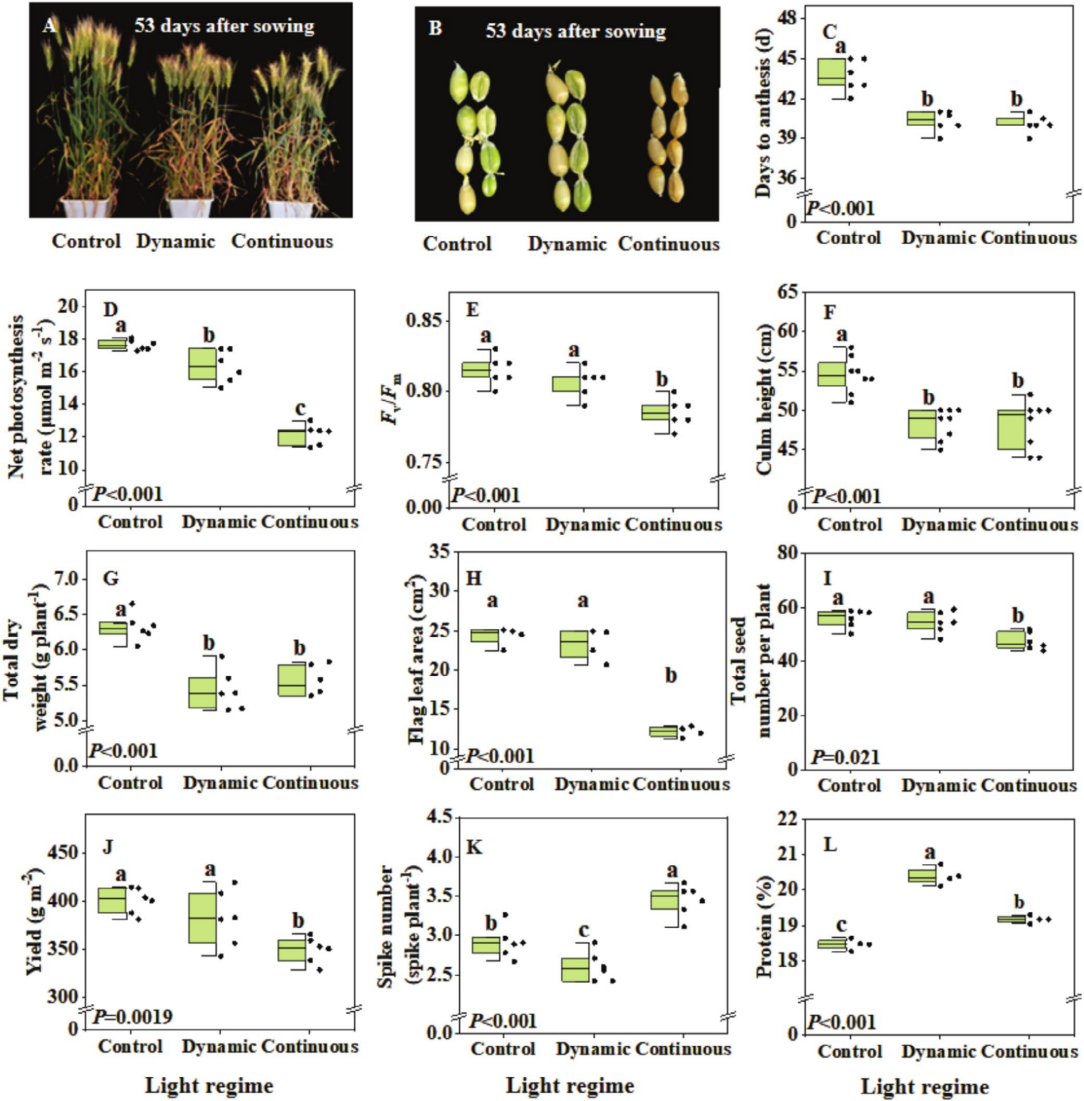
Effects of light regimes on growth and development of wheat. (A) Image of plants at 53 days after sowing; (B) image of seeds at 53 days after sowing; (C) days from sowing to anthesis (*n* = 6); (D) net photosynthesis rate (*n* = 6); (E) maximum photochemical efficiency of photosystem II (*F*_v_/*F*_m_) (*n* = 6); (F) culm height (*n* = 8); (G) total dry weight (including stem and spike) (*n* = 6); (H) flag leaf area (*n* = 4); (I) total seed number per plant (*n* = 6); (J) wheat yield (*n* = 6); (K) spike number per plant (*n* = 6); (L) seed protein content (*n* = 4). Plants were grown in substrate cultivation under three different light regimes with similar daily light integral: (1) Control: 500 μmol m^−2^ s^−1^ PPFD with 22 h photoperiod; (2) Continuous: 458 μmol m^−2^ s^−1^ PPFD with 24 h photoperiod (continuous light); (3) Dynamic: Different light intensities used during different developmental stages, at a photoperiod of 22 h (Table 3). Box plots indicate 0% (maximum), 25%, 50% (median), 75%, and 100% (minimum), and points show single data. Different letters represent significant differences between treatments according to Duncan's analysis at *p* = 0.05.

Images of plant growth and seed maturity at different growth stages were shown in Figure 4A,B. Wheat flowered 4 days earlier in both dynamic and continuous light treatments compared to the control (Figure 4C), but both the dynamic and the continuous light treatments decreased net leaf photosynthesis rate, *F*_v_/*F*_m_, and chlorophyll index compared to the control (Figure 4D,E; Table S3). The continuous light treatment not only reduced culm height and total dry weight compared to the control (Figure 4F,G), but also reduced the flag leaf area, total seed number, final yield, and grain number per spike compared to the control and dynamic light treatments (Figure 4H–J; Table S3). However, the continuous light treatment increased spike number by 19% and 33% compared to the control and dynamic light treatments, respectively (Figure 4K). The dynamic light treatment could significantly increase seed protein content compared to other treatments (Figure 4L).

### 3.4 Effects of Color Temperature on Spring Wheat Growth and Development

**FIGURE 5:**
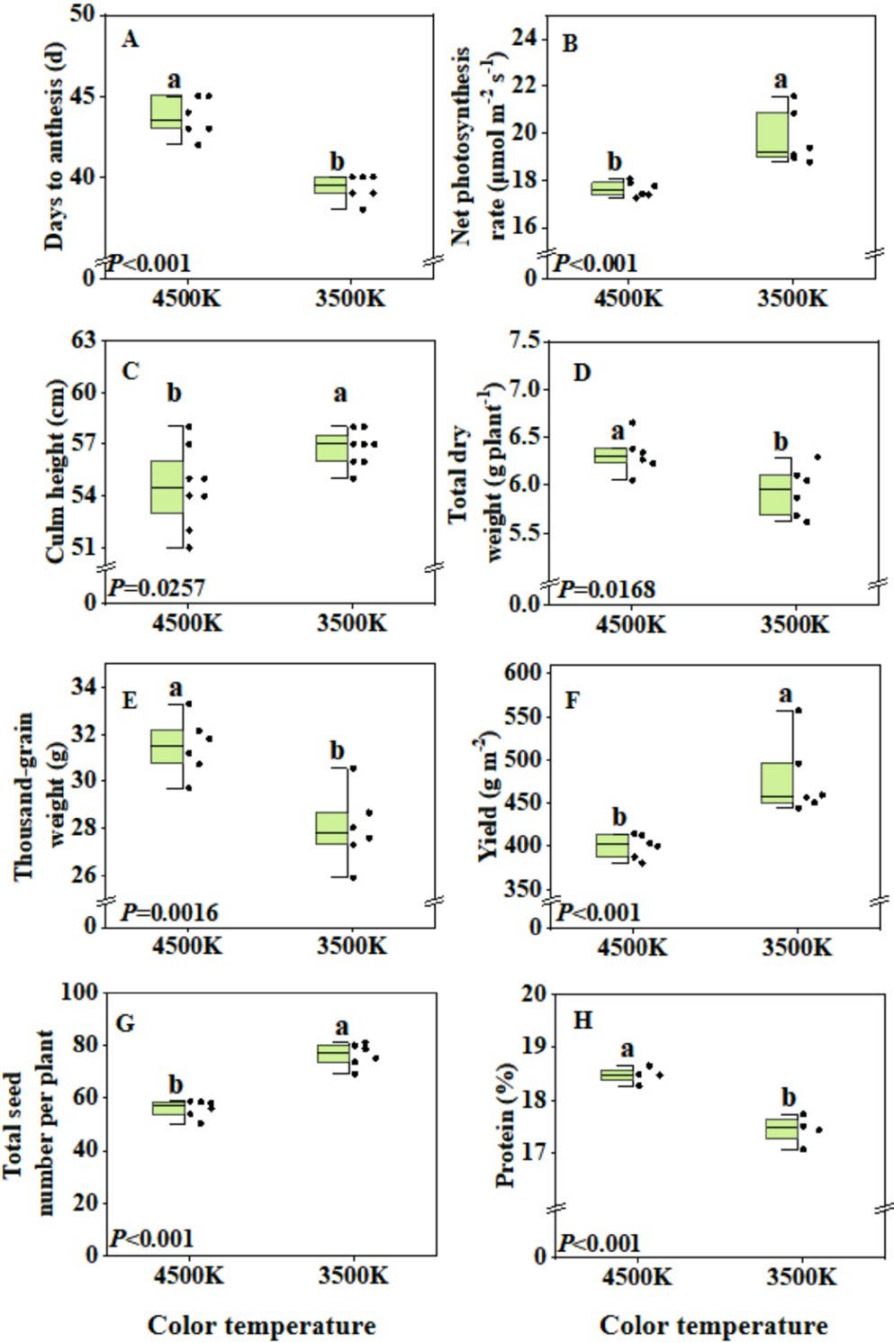
Effects of color temperature on spring wheat growth and development. (A) Days from sowing to anthesis (*n* = 6); (B) net photosynthesis rate (*n* = 6); (C) culm height (*n* = 8); (D) total dry weight (including stem and spike) (*n* = 6); (E) thousand‐grain weight (*n* = 6); (F) wheat yield (*n* = 6); (G) total seed number per plant (*n* = 6), (H) seed protein content (*n* = 4). Box plots indicate 0% (maximum), 25%, 50% (median), 75%, and 100% (minimum), and points show single data. Different letters for each column represent significant differences between treatments according to Duncan's analysis at *p* = 0.05.

Growth under 3500 K light caused wheat to flower 5 days earlier, while increasing net leaf photosynthesis rate by ~12% and culm height compared to 4500 K (Figure 5A–C). Although 3500 K decreased total dry weight and thousand‐grain weight, it increased yield by ~14% compared to the 4500 K treatment. The 3500 K light treatment also increased dry matter partitioning to spikes, total seed number, and grain number per spike (Figure 5D–G; Table S4). Furthermore, the 3500 K light treatment decreased seed protein content compared to the 4500 K light treatment (Figure 5H).

### 3.5 Effects of Harvest Time on Seed Germination

**FIGURE 6:**
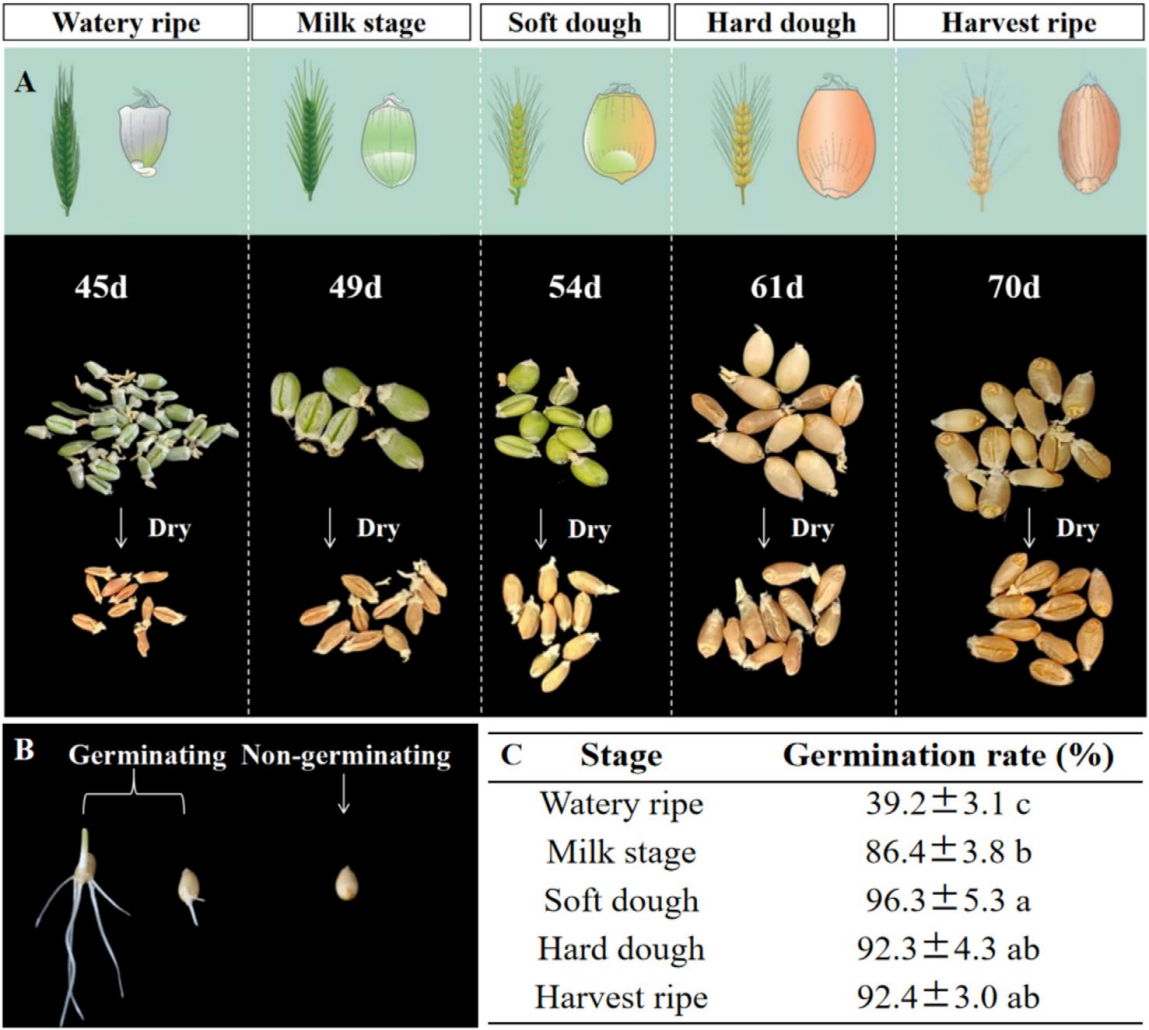
Seed germination rate at different stages of maturity. (A) seeds harvested at different developmental stages (no. days after sowing) and dried at 38°C for 2 days until water content reached ~15%; (B) image of seed germination; (C) seed germination rate. Data are presented as means ± SE (*n* = 3, where each replicate represents the mean of thirty biological replicates). Means were compared using ANOVA, and different letters represent significant differences between treatments according to Duncan's analysis at *p* = 0.05.

Seeds harvested at the watery ripe stage showed a significantly lower germination rate than those harvested at other stages of maturity (Figure 6). Beyond the soft dough stage, the germination rate reached > 90% (Figure 6C).

## 4 Discussion

We show here that the light environment (spectrum, intensity, photoperiod as well as dynamic pattern) played a key role in wheat growth, development, leaf photosynthetic properties, as well as yield and seed quality. Below, we discuss how to promote flowering and seed production of spring wheat in indoor farming by manipulating the light environment. Further, we provide perspectives on future indoor wheat cultivation.

### 4.1 Speed Flowering in Wheat: High Light Intensity, Continuous Lighting and Reduction in the Proportion of Blue Light

Early flowering is a key trait for speed breeding (Kabade et al. 2024) and plants can sense light signals by photoreceptors to regulate flowering (Bao et al. 2024). Several studies showed that a PPFD of 400–500 μmol m^−2^ s^−1^ at a 22 h photoperiod was suitable for speedy flowering in wheat (Dong et al. 2014; Guo et al. 2025, 2023). We found that by further increasing PPFD (to 700–900 μmol m^−2^ s^−1^), wheat can flower 5–10 days earlier compared to 300–500 μmol m^−2^ s^−1^ (Figure 3C). So far, the effects of light on flowering have mainly been viewed through photoperiod and light quality (Maple et al. 2024), while the effects of light intensity have been studied less well. One may speculate that high PPFD could induce early flowering by enhancing the accumulation of assimilates (such as sucrose), which act as an energy and/or signaling molecule for flowering (Srikanth and Schmid 2011). However, when increasing PPFD from 700 to 900 μmol m^−2^ s^−1^, the leaf net photosynthesis rate decreased by 13%, while the flowering time did not change (Figure 3C,E). In *Arabidopsis thaliana* (Arabidopsis), Feng et al. (2016) revealed that high light‐regulated flowering involves retrograde signaling from the chloroplast to the nucleus and the activity of FLOWERING LOCUS C (FLC). Hence, retrograde signaling due to high light intensity may induce flowering in spring wheat grown under PPFD > 700 μmol m^−2^ s^−1^.

Continuous light (no dark period) was found to speed up flowering compared with a 22 h photoperiod (Figure 4C). The flowering response to changes in the photoperiod is mediated by complex interactions between environmental signals and the circadian clock (Lee et al. 2023). The circadian clock is an autonomous regulator that generates endogenous rhythms with a period of approximately 24 h, including expression of a transcriptional regulator CONSTANS (CO) (San Martin and Yanovsky 2024). In Arabidopsis, which is also a long‐day plant, CO can be activated under long days and can induce the expression of FLOWERING LOCUS T (FT), a gene that promotes flowering (Kamran et al. 2014; Valverde 2011), and whose mechanism has been confirmed in wheat (Li et al. 2024). In Arabidopsis, Jang et al. (2008) found that the CO protein was degraded in darkness by CONSTITUTIVE PHOTOMORPHOGENIC 1/SUPPRESSOR OF PHYTOCHROME A‐105 (COP1/SPA) complex(es). Therefore, we speculate that a continuous 24‐h illumination blocks COP1/SPA‐mediated CO degradation and sustains CO accumulation, which could induce FT to promote flowering in wheat, but this requires further investigation.

When the fraction of blue light in full‐spectrum white LEDs increased from 21% to 65% (Table 1), flowering was delayed by 12 days (Figure 2B), which was highly consistent with a previous study showing that a high blue light fraction delayed wheat flowering (Monostori et al. 2018). This was also supported by the results of our color temperature experiment: compared with cold white LEDs (4500 K, 21% blue light), warm white LEDs (3500 K, 14% blue light) contained less blue light and resulted in a shortened flowering time by 4 days (Figure 5A). In Arabidopsis, cryptochrome 2 (CRY2) is a key inducer of flowering under long‐day conditions. In this situation, CRY2 can stabilize the floral promoter CO via suppression of the COP1/SPA complex (Cao et al. 2021; Ponnu 2020; Ponnu and Hoecker 2022). However, CRY2 responds primarily to dim blue light and is degraded within a few hours of exposure to a higher fluence rate of blue light (Liu et al. 2022). Therefore, we speculate that the increased blue light fraction could delay flowering in wheat due to a reduced CRY2 activation. Future studies may elucidate how strong blue light delays wheat flowering through cryptochrome pathways as well as the coaction in modulating COP1/SPA activity.

### 4.2 Speed Breeding Does Not Necessarily Come at the Cost of Seed Production

Intuitively, one might think that the ideal light recipe for speed breeding causes a loss of seed production, as it results in a reduction of the growth period. However, we found that suitable light intensity and spectrum did not only shorten the generational cycle, but also maintained high yields. For example, compared to a light color of 4500, 3500 K not only induced early flowering but also increased seed yield (Figure 5A,F), largely due to increased biomass partitioning to the spikes (Table S4), while total shoot biomass was slightly reduced (Figure 2D). These results suggest that light spectrum (particularly red and blue fraction, Table 2) may regulate carbohydrates partitioning in spring wheat. Numerous studies have shown that sucrose is the primary form in which carbohydrates are transported from source to sink organs, a process dependent on sucrose transporters such as SWEET proteins (Chen 2014; Peixoto et al. 2021). However, the mechanisms by which red and blue light proportions alter sucrose transport in wheat remain to be clarified.

As for light intensity, under 22 h photoperiod, yield peaked and plants entered anthesis earliest under 700 μmol m^−2^ s^−1^ (Figure 3C). The increase in yield was mostly due to a high leaf photosynthesis rate, large spike number per plant, and grain number per spike (Figure 3E,L; Table S2). Although an increase in PPFD from 700 to 900 μmol m^−2^ s^−1^ increased the spike number per plant further (Figure 3L), it strongly reduced the leaf photosynthesis rate and caused *F*_v_/*F*_m_ to be less than 0.8 (Figure 3D,E), indicating photoinhibition under 900 μmol m^−2^ s^−1^. Therefore, high PPFD may destroy D1 protein in the photosystem II reaction center, thus reducing electron transfer efficiency and photosynthesis (Chen et al. 2020).

Although continuous light can shorten the flowering of wheat, it results in a reduced yield compared to a photoperiod of 22 h (Figure 4C,G). The decrease in leaf chlorophyll content as well as *F*_v_/*F*_m_ indicated an occurrence of photoinhibition under continuous light (Figure 4E; Table S3). Besides, decreased shoot biomass partitioning to spikes under continuous light (Figure 4E; Table S3) further reduced seed production compared to 22 h. A previous study in tomato found that increased intensity of far‐red (700–800 nm) light can mitigate the adverse effects of continuous light through the phytochrome pathway (Velez‐Ramirez et al. 2019). Future studies are needed to reduce adverse effects of continuous light on plant growth and yield through light spectrum manipulation in spring wheat.

### 4.3 Light Recipes and Perspectives on Speed Breeding of Spring Wheat in Indoor Farming

**FIGURE 7:**
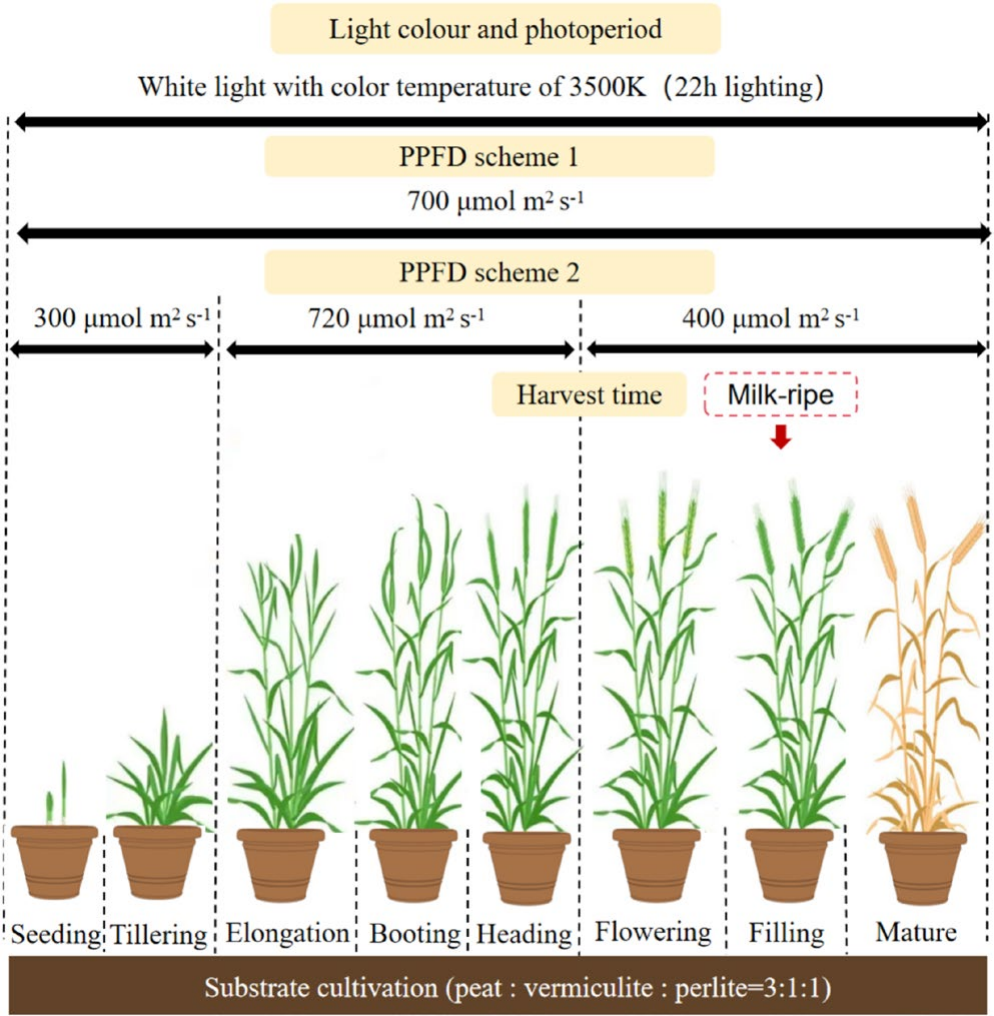
Summary of cultivation environment and lighting strategy for spring wheat grown in indoor farming. PPFD scheme 1, constant light intensity. PPFD scheme 2, dynamic light intensity.

Indoor cultivation techniques have shown significant advantages in wheat breeding in the past few years (Guo et al. 2023). Here we summarize two lighting recipes for growing spring wheat in indoor farming (Figure 7). As for constant PPFD without considering energy use, we propose 700 μmol m^−2^ s^−1^ at a photoperiod of 22 h; this causes anthesis within 35 days and fully ripe seeds in 55 days, while achieving high yields. It is worth noting that there are likely differences in light demand at different developmental stages of wheat (Slafer et al. 2023). Before the tillering stage, plants may have a relatively low demand for photosynthetic products, and maintaining a medium PPFD (e.g., 300–500 μmol m^−2^ s^−1^, 22 h) likely meets the growth requirement (Dong et al. 2014). After entering the jointing stage, the spike differentiation process is accelerated, and a large number of assimilates are needed to support the establishment of reproductive organs, which is key for sink strength determination during grain filling (Zhu et al. 2019). Therefore, we also propose a dynamic light regime, which can save ~30% of light input compared to constant PPFD of 700 μmol m^−2^ s^−1^, at only minor costs to yield (11%). Our experiment on comparing a dynamic with a constant light regime (Figure 4) serves as a preliminary proof of concept.

Apart from visible light, recent studies revealed that the application of far‐red light can induce flowering in wheat by activating phytochrome signaling pathways (Kong and Zheng 2025; Shomali et al. 2024). This came at the cost of inhibited tiller formation, thus reducing panicle formation and yield (Lei et al. 2022; Ugarte et al. 2010). However, it is still worth exploring whether low‐intensity far‐red light (< 50 μmol m^−2^ s^−1^) that is applied before tillering can advance flowering time without decreasing yield.

Besides light, speed breeding may be facilitated with other environmental control as well as cultivation practices. Gradually reducing soil moisture content can facilitate grain filling and seed maturation (Watson et al. 2018). The use of immature seeds for propagation can further minimize generation time (Ghosh et al. 2018; Xu et al. 2022). For example, seeds harvested at the watery ripe stage (44 days after sowing) did germinate (Figure 6C), indicating the possibility of eight generations per year (Figure 6A–C). Early harvesting combined with advanced young embryo culture could further shorten the generational cycle (Zheng et al. 2013). However, whether using immature seeds reduces the quality or yield of subsequent generations or not has not been verified. Also, increasing air temperature to 5°C above ambient air temperature was shown to shorten the generational cycle in wheat by 12 days, but at a cost of yield loss (Asseng et al. 2011; Farooq et al. 2011). To optimize economic and resource use efficiency, it is necessary to continuously adjust environmental variables (e.g., temperature, humidity, CO_2_ concentration, etc.) and to have accurate sensors to tell whether the crop is making optimal use of those environmental factors. Integrating AI and genome‐editing technologies will allow for largely improved breeding efficiency and the precise design of crops tailored to specific growth conditions (Baiyin and Yang 2024). As for future wheat production in indoor farming, food quality and safety are just as important as yield. As seed starch and protein contents have long been measured nondestructively by detecting spectral properties, spectral detection models for other seed qualities (e.g., dietary fiber, vitamins, phytochemicals, minerals, etc.) may also be developed (Rahman and Cho 2016).

## 5 Conclusions

A full spectrum of white LEDs (3500 K) was found to be a promising spectrum for wheat growth. As long‐day crops, spring wheat can endure 24 h constant light, but a 20–22 h photoperiod was found to be more optimal. We propose that a PPFD of 700 μmol m^−2^ s^−1^ under a 22 h photoperiod can trigger anthesis within 35 days, while achieving a high yield. However, considering electricity consumption, we further recommended a dynamic light intensity strategy (~300 μmol m^−2^ s^−1^ from seedling to tillering stage; ~700 μmol m^−2^ s^−1^ during elongation to heading stage; ~400 μmol m^−2^ s^−1^ after flowering stage), which can save ~30% of light input compared to a constant PPFD of 700 μmol m^−2^ s^−1^, but only at a small yield loss (11%). Seeds can be harvested at the milky stage to further reduce the seed‐to‐seed cycle. With these measures, the generation cycle may be compressed to < 50 days, achieving more than seven generations per year in spring wheat.

## Ethics Statement

The authors have nothing to report.

## Consent

The authors have nothing to report.

## Conflicts of Interest

The authors declare no conflicts of interest.

## Supporting information


**Table S1.–S4.**pei370085‐sup‐0001‐Supinfo.pdf.

## Data Availability

The data that support the findings of this study are openly available in Mendeley Data at https://data.mendeley.com/datasets/j3kzmd4cft/1.
