# Supplementary material for: Light Spectrum, Intensity, and Photoperiod Are Key for Production as Well as Speed Breeding of Spring Wheat in Indoor Farming
Source: Plant Environ Interact. 2025 Sep 10;6(5):e70085. doi: 10.1002/pei3.70085 (PMC12421069; doi:10.1002/pei3.70085)
Supplement: Supplementary file 1 — Table S1.–S4.pei370085‐sup‐0001‐Supinfo.pdf. [file PEI3-6-e70085-s001.docx]

**Supplementary Information**

| **Table S 1**. Effects of light quality on spring wheat growth and production | | | | |
| --- | --- | --- | --- | --- |
| Parameter | Light quality | | | |
|  | W | W + R | W + G | W + B |
| Culm height (cm) | 50.7±1.8 c | 58.4±3.3 b | 53.0±3.0 c | 61.6±4.8 a |
| Flag leaf area (cm^2^) | 30.5±0.3 b | 42.1±0.3 a | 23.2±0.2 c | 21.8±0.2 c |
| Dry matter partitioning to spike (%) | 44.4±1.9 a | 37.4±1.5 bc | 39.9±3.8 b | 35.7±3.9 c |
| Dry matter partitioning to stem (%) | 55.6±1.9 c | 62.6±1.5 ab | 60.2±3.8 b | 64.30±3.9 a |
| Grain number per spike (grain spike^-1^) | 15.7±0.7 b | 17.9±1.3 a | 14.8±0.7 b | 17.6±0.8 a |
| Thousand-grain weight (g) | 23.8±0.5 a | 22.2±1.1 bc | 21.5±1.0 c | 22.9±0.5 ab |
| Protein (%) | 18.8±0.2 b | 18.7±0.1 b | 19.7±0.1 a | 18.7±0.2 b |
| Starch (%) | 73.6±0.4 ab | 73.5±0.2 ab | 72.8±0.1 b | 73.7±0.4 a |
| Days to heading (d) | 47.5±2.4 c | 51.4±3.5 b | 47.6±1.3 c | 56.7±1.5 a |
| Days to mature (d) | 70.3±2.5 c | 75.2±1.6 b | 71.8±2.4 c | 85.3±2.6 a |
| Note: Wheat was grown under four different light quality treatments: white (W), white + green (W+G), white + blue (W+B), white + red (W+R) at PPFD of ~300 μmol m^-2^ s^-1^ with 20 h photoperiod. Data are presented as means ± SE of six to twelve independent samples. Means were compared using ANOVA, and different letters indicate statistically significant differences between layers. (PPFD-photosynthetic photon flux density). | | | | |

| **Table S 2**. Effects of light intensity on spring wheat growth and production | | | | |
| --- | --- | --- | --- | --- |
| Parameter | PPFD (μmol m^-2^ s^-1^) | | | |
|  | 300 | 500 | 700 | 900 |
| Flag leaf area (cm^2^) | 20.1±0.7 a | 14.8±0.8 b | 10.9±1.0 c | 7.8±0.4 d |
| Dry matter partitioning to spike (%) | 41.2±0.8 b | 48.0±1.8 a | 46.5±3.5 a | 45.5±2.4 a |
| Dry matter partitioning to stem (%) | 58.8±0.8 a | 52.0±1.8 b | 53.5±3.5 b | 54.5±2.4 b |
| *F*_v_/*F*_m_ | 0.77±0.01 c | 0.82±0.01 a | 0.83±0.01 a | 0.79±0.01 b |
| Grain number per spike (grain spike^-1^) | 18.2±0.9 c | 24.3±1.3 b | 28.8±1.1 a | 19.6±0.8 c |
| Protein (%) | 17.9±0.2 ab | 17.5±0.1 bc | 18.0±0.4 a | 17.4±0.1 c |
| Starch (%) | 74.3±0.2 b | 74.7±0.1 ab | 74.2±0.5 b | 74.9±0.3 a |
| Days to heading (d) | 40.3±2.4 a | 35.2±2.2 b | 31.0±2.8 c | 30.2±1.6 c |
| Days to mature (d) | 68.0±3.2 a | 62.8±2.7 b | 55.2±3.4 c | 56.1±3.1 c |
| Note: Plants were grown in substrate cultivation at PPFD of ~300 μmol m^-2^ s^-1^; 500 μmol m^-2^ s^-1^; 700 μmol m^-2^ s^-1^; 900 μmol m^-2^ s^-1^ with 22 h photoperiod. Data are presented as means ± SE of six to twelve independent samples. Means were compared using ANOVA, and different letters indicate statistically significant differences between layers. (PPFD-photosynthetic photon flux density) | | | | |

| **Table S 3**. Effects of light regimes on growth and production of wheat. | | | |
| --- | --- | --- | --- |
| Parameter | Lighting regimes | | |
|  | Control | Dynamic | Continuous |
| Specific flag leaf area (cm^2^ g^-1^) | 264.1±10.2 a | 240.6±6.2 b | 241.1±10.0 b |
| Dry matter partitioning to spike (%) | 28.5±1.4 b | 31.2±1.3 a | 27.6±2.9 b |
| Dry matter partitioning to stem (%) | 71.5±1.4 a | 68.8±1.3 b | 72.4±2.9 a |
| Grain number per spike (grain spike^-1^) | 19.2±1.4 a | 21.0±1.6 a | 14.5±1.4 b |
| Thousand-grain weight (g) | 31.5±1.1 a | 30.9±0.8 a | 30.2±1.2 a |
| Chlorophyll index | 1.3±0.1 a | 1.1±0.1 b | 0.9±0.1 c |
| Protein (%) | 19.2±0.1 b | 18.5±0.1 c | 20.4±0.2 a |
| Starch (%) | 73.5±0.1 b | 74.2±0.2 a | 72.4±0.2 c |
| Days to heading (d) | 39.2±1.1 a | 34.1±1.8 b | 33.5±2.1 b |
| Days to mature (d) | 66.3±2.2 a | 59.2±1.7 b | 58.1±1.6 b |
| Note: Plants were grown in substrate cultivation under three different light regimes with similar daily light integral: 1) Control: 500 μmol m^-2^ s^-1^ PPFD with 22 h photoperiod; 2) Continuous: 458 μmol m^-2^ s^-1^ PPFD with 24 h photoperiod (continuous light); 3) Dynamic: different light intensities used during different developmental stages, at a photoperiod of 22 h (Table 3). Data are presented as means ± SE of six to twelve independent samples. Means were compared using ANOVA, and different letters indicate statistically significant differences between layers. (PPFD-photosynthetic photon flux density) | | | |

| **Table S 4**. Effects of color temperature on spring wheat growth and production. | | |
| --- | --- | --- |
| Parameter | color temperature | |
|  | 4500 K | 3500 K |
| Flag leaf area (cm^2^) | 24.3±1.0 a | 24.0±1.4 a |
| Specific flag leaf area (cm^2^ g^-1^) | 264.1±10.2 a | 267.5±14.1 a |
| Dry matter partitioning to spike (%) | 37.7±1.9 a | 25.5±1.4 b |
| Dry matter partitioning to stem (%) | 62.3±1.9 b | 74.5±1.4 a |
| Spike number (spike plant^-1^) | 2.9±0.2 a | 2.9±0.1 a |
| Grain number per spike (grain spike^-1^) | 18.9±1.2 b | 27.8±2.3 a |
| Protein (%) | 18.5±0.1 a | 17.4±0.2 b |
| Starch (%) | 74.2±0.2 a | 75.4±0.2 a |
| Days to heading (d) | 40.2±1.2 a | 35.3±1.8 b |
| Days to mature (d) | 66.3±2.2 a | 59.6±1.8 b |
| Note: Plants were grown in substrate cultivation at PPFD of 500 μmol m^-2^ s^-1^ with 22 h photoperiod. Data are presented as means ± SE of six to twelve independent samples. Means were compared using ANOVA, and different letters indicate statistically significant differences between layers. (PPFD-photosynthetic photon flux density) | | |
